# Incidence and Prognostic Significance of Silent Coronary Disease in Asymptomatic Patients with Severe Aortic Stenosis

**DOI:** 10.3390/medicina60091503

**Published:** 2024-09-14

**Authors:** Marko Cubrilo, Marko Banovic, Milos Matkovic, Ilija Bilbija, Nemanja Aleksic, Dragan Ivanisevic, Vladimir Tutus, Vladimir Milicevic, Vladimir Cvetic, Natasa Jankovic, Svetozar Putnik

**Affiliations:** 1 Department for Cardiac Surgery, University Clinical Centre of Serbia, 11000 Belgrade, Serbiai.bilbija@yahoo.com (I.B.);; 2 Faculty of Medicine, University of Belgrade, 11000 Belgrade, Serbia; markobanovic71@gmail.com (M.B.); drvladimircvetic@gmail.com (V.C.); 3 Department for Cardiology, University Clinical Centre of Serbia, 11000 Belgrade, Serbia; 4 Department for Anaesthesiology, Reanimation and Intensive Care, University Clinical Centre of Serbia, 11000 Belgrade, Serbia; 5 Department for Cardiovascular Radiology, University Clinical Centre of Serbia, 11000 Belgrade, Serbia

**Keywords:** severe aortic stenosis, silent coronary disease, asymptomatic

## Abstract

*Background and Objectives*: The aim of this study was to estimate the prevalence of silent coronary artery disease (CAD) in asymptomatic patients with severe aortic stenosis (AS) and assess long-term prognosis in terms of major adverse cardiovascular event (MACE)-free survival. *Materials and Methods*: This was a prospective study conducted at the Clinic for Cardiac Surgery, University Clinical Center of Serbia, in asymptomatic patients with severe AS, normal LVEF and stress test without signs of myocardial ischemia. Adverse cardiovascular events (cardiac death, myocardial infarction and any hospitalization due to heart disease) was monitored during one year of follow up. *Results*: A total of 116 asymptomatic patients with severe AS were included in the study. The average age was 67.3 ± 9.6 years, and 56.9% of patients were men. The most common cause of AS was degenerative valvular disease (83.5%). The incidence of significant CAD was 30 out of 116 patients (25.9%). The median Society for Thoracic Surgeons (STS) predicted risk of mortality score was 1.62% (25th to 75th percentile: 1.15–2.76%). The overall mean gradient across aortic valve (Pmean) was 52.30 mmHg ± 12.16, and the mean indexed AVA (AVAi) was 0.37 ± 0.09 cm^2^/m^2^. The mean LVEF was 68.40% ± 8.01%. Early surgery for aortic valve replacement was performed in 55 patients (55.2%), while 52 (44.8%) patients received conservative treatment. Twenty-two patients (42.3%) in the conservative treatment group underwent surgery during follow up. There were a total of 44 (37.9%) patients with MACE during one year of follow up. Univariate Cox regression analyses identified the following significant risk factors for MACE-free survival: presence of CAD and early conservative treatment (*p* = 0.004), age (*p* = 0.003), diabetes mellitus (*p* = 0.016) and STS score (*p* = 0.039). According to multivariate analysis, the presence of CAD with early conservative treatment was the most important predictor of MACE-free survival in asymptomatic patients with severe aortic stenosis (*p* ≤ 0.001). *Conclusions*: Early surgery for aortic valve replacement in asymptomatic patients with severe AS and concomitant CAD is beneficial for long-term survival.

## 1. Introduction

Aortic stenosis (AS) is the most common valvular heart disease in developed countries [1,2]. While surgical aortic valve replacement, or percutaneous replacement as an alternative, is indicated in patients with severe symptomatic AS [1,2,3], there is still no consensus on the treatment of patients with severe hemodynamically significant asymptomatic AS. Consequently, patients with similar characteristics may undergo surgery in one center, while in another, they are conservatively monitored until symptoms appear before undergoing surgery. This being the case, recent research has brought to light new data that have swayed the consensus into a single direction. Banovic et al. concluded the AVATAR Trial [4] (Aortic Valve Replacement Versus Conservative Treatment in Asymptomatic Severe Aortic Stenosis) by showcasing results suggesting that surgical aortic valve replacement should indeed be undergone by patients with AS.

The data indicate that around 50% of symptomatic patients with severe AS also have coronary artery disease [5]. However, what is not known is the percentage/prevalence of asymptomatic patients with significant AS who have “hidden/latent coronary artery disease.” The only relevant data could be found in a small-scale study, and Banovic et al. suggest that 19.8% of patients had significant coronary artery disease on coronary angiography [6], encompassing 96 patients in short-term follow up. This is highly significant because associated coronary artery disease strengthens the indication for surgery to the extent that it is recommended to replace the aortic valve even in the presence of moderate AS if the patient requires surgical myocardial revascularization. Additionally, unrecognized coronary artery disease alongside existing hemodynamically significant AS could increase the risk of ischemia, malignant arrhythmias and adverse cardiovascular events. Nevertheless, there is a lack of data on the frequency of hidden/latent (without symptoms or signs of ischemia) coronary artery disease in asymptomatic patients with severe AS. Exercise testing plays a significant role not only in assessing the symptomatic status of patients with AS but also in evaluating the risk of future adverse events [1,2]. However, exercise tests in patients with significant valvular defects are not sufficiently utilized in everyday clinical practice, perhaps due to the ongoing concern held by a considerable number of physicians about potential adverse events during testing. Therefore, there are no clear data on the frequency of silent coronary artery disease in asymptomatic patients with severe AS and normal left ventricular ejection fraction (LVEF), nor on the potential predictors of masked coronary artery disease. Additionally, there are no comparative data on the frequency of adverse events in asymptomatic patients with severe AS and hidden coronary artery disease compared to patients without coronary artery disease. Therefore, the aim of this study was to estimate the prevalence of silent CAD in asymptomatic patients with severe aortic stenosis and assess their long-term prognosis in terms of MACE-free survival.

## 2. Materials and Methods

### 2.1. Study Design

This was a prospective study conducted at the Department of Cardiac Surgery, University Clinical Center of Serbia, in the period beginning 1 January 2021 and included asymptomatic patients with AS, normal LVEF and stress test without signs of myocardial ischemia. Each patient underwent a one-year follow up, during which the occurrence of adverse cardiovascular events (cardiac death, myocardial infarction and any hospitalization due to heart disease) was monitored and recorded. Asymptomatic patients selected for surgical aortic valve replacement underwent an echocardiographic examination, stress test and (classical/MSCT) coronary angiography. The study was approved by Ethics Committee of the University Clinical Center of Serbia (NCT02436655), and informed consent was obtained from all patients. 

### 2.2. Inclusion and Exclusion Criteria

The main criteria for the enrollment of a patient in the study was the existence of AS, verified using echocardiography, along with the absence of any reported symptoms. 

Echocardiographic criteria for the presence of narrow aortic stenosis included the following:

(a) Maximum flow speed (vmax) > 4 m/s;
(b) Mean gradient across the aortic valve ≥ 40 mmHg;
(c) Area of the aortic orifice < 1 cm^2^.

General criteria for exclusion from the study were reported symptoms, associated moderate or significant valvular defect, LVEF < 50%, the presence of associated aortic disease, significant renal insufficiency (eGFR < 30 mmmol), significant obstructive lung disease (FEV1 ≤ 70%), previous cardiac surgery, known coronary disease and estimated life expectancy <3 years. 

### 2.3. Procedures

All patients underwent a detailed history as well as a clinical workup that included physical examination, laboratory analysis, echocardiographic examination of the heart, spirometry, stress test and coronary angiography.

Laboratory analysis: For the laboratory analyses, routine biochemical analyses was performed on each patient: complete blood count, glycemia, bilirubin, urea, creatinine, albumin, cholesterol, triglycerides, HDL (high-density lipoprotein), LDL (low-density lipoprotein), aspartate aminotransferase (AST), alanine aminotransferase (ALT), alkaline phosphatase, gamma-glutamyl transpeptidase (gGT), lactate dehydrogenase (LDH), partial thromboplastin time (PTT), prothrombin time (PT), INR (international normalized ratio), fibrinogen and C-reactive protein (CRP). Brain natriuretic peptide (BNP) and NT-proBNP markers were examined to confirm the asymptomatic status of the patients, given that the current European guidelines for the treatment of valvular heart disease suggest aortic valve replacement in asymptomatic patients with severe aortic stenosis who have blood natriuretic peptide levels three times higher than normal. 

Echocardiography: Echocardiography was performed using a Vivid 4 machine (BTO6, 1.5–3.6 MHz; GE Healthcare Technologies, Waukesha, WI, USA). Patients were examined in the left lateral position using parasternal longitudinal and transverse sections as well as 4-, 2- and 3-cavity apical sections.

The maximum pressure across the aortic valve (Pmax) was calculated from the maximum velocity of the blood flow using the simplified Bernoulli equation: Pmax = 4Vmax2. The mean gradient was calculated by approximating the current gradients within a certain time interval 5.38: Pmean = (4V12 + 4V22 + … 4Vn2)/n. 

The aortic orifice area (AVA) was calculated based on the continuity equation AVA = CSAlvot x (VTIlvot/VTIAo), where CSA represents the diameter of the LV outflow tract, while VTIlvot and VTIAo represent the velocity–time integrals in the LV outflow tract and over the aortic valve itself, which are obtained by pulsed, i.e., continuous, Doppler recording.

Ejection fraction and the end-diastolic and end-systolic LV volumes were determined using 2D echocardiography according to Simpson’s model. Mitral flow was analyzed by pulse Doppler at the level of the tips of the mitral leaflets. The grading of possible mitral (MR) and aortic insufficiency (AR) was conducted using color and continuous Doppler, while the assessment of the degree of tricuspid regurgitation (TR) and systolic pressure in the right ventricle (SPDK) was performed based on the pressure gradient (PG) on the tricuspid mouth and the dimensions and respiratory mobility of the inferior vena cava. Tissue Doppler (TDI), as a method for quantifying regional myocardial function, enabled the analysis of the myocardial velocities of selected segments of the LK. The ratio of the mitral E wave to the E’ wave of the mitral annulus (E/Ea) correlates with left atrial pressure and pulmonary capillary pressure and is important for diagnosing diastolic dysfunction.

Coronary angiography: Coronary angiography was performed percutaneously/invasively. Percutaneous coronary angiography was performed using a standard radial or femoral approach. Any epicardial coronary stenosis was visually evaluated from multiple projections. Any stenosis that was > 70% in one of the main arteries or > 50% in the main left coronary artery was considered significant. A significance of stenosis ≤ 50 ≤ 70% in one of the 3 main heart arteries was assessed using the FFR (fractional flow reserve) method. A value ≤ 0.8 was considered significant.

### 2.4. Statistical Analysis

Numerical data were presented as mean with standard deviation or 95% CI or as median with 25th and 75th percentiles. Categorical variables were summarized as absolute numbers with percentages. The normality of the data was assessed using mathematical (Kolmogorov–Smirnov, skewness and kurtosis, coefficient of variation) and graphical (histogram, box plot) methods. Differences between groups were assessed using the Student’s *t*-test, Mann–Whitney U test and Chi-square test. Kaplan–Meier curves and Cox hazard regression analysis was used for survival analysis. Statistically significant variables (*p* < 0.05) in univariate analysis were included in multivariate regression analysis in order to assess the predictors of MACE-free survival. In all analyses, the significance level was set at 0.05. Statistical analysis was performed using IBM SPSS statistical software (SPSS for Windows, release 25.0, SPSS, Chicago, IL, USA).

## 3. Results

A total of 116 asymptomatic patients with severe AS were included in the study. The average age of enrolled patients was 67.3 ± 9.6 years, with 56.9% males and 43.1% females. The cause of AS was degenerative valvular disease in 83.5% patients and bicuspid aortic valve in 16.5% of patients. Significant CAD on CA was found in 30/116 patients (25.9%). CAD was present in the right coronary artery in 15.5% patients, left coronary artery in 11.2% and Cx in 11.2% of patients. Ten patients (8.6%) had more than one artery affected.

The median Society for Thoracic Surgeons predicted risk of mortality score was 1.62% (25th to 75th percentile: 1.15–2.76%). The overall mean gradient across the aortic valve (Pmean) was 52.30 mmHg ± 12.16, and the mean indexed AVA (AVAi) was 0.37 ± 0.09 cm^2^/m^2^. The mean LVEF was 68.40% ± 8.01%, with a minimum value of 40% and maximum value of 82%. Most patients had hypertension (91.2%), while 24.3% had diabetes mellitus. Heredity was present in 6.1% of patients, and 22.6% were smokers/ex-smokers.

Early surgery for aortic valve replacement was performed in 55 patients (55.2%), while 52 (44.8%) patients received conservative treatment. Twenty-two patients (42.3%) in the conservative treatment group underwent surgery during follow up. The median time to surgery in the conservative treatment group was 402 days (25th to 75th percentile: 179–674). Furthermore, 50.0% of patients received a mechanical valve, 44.6% received a bioprosthetic valve and 5.4% received a sutureless bioprosthetic valve. Demographic characteristics, cardiovascular risk factors and medical therapy, as well as baseline laboratory and echocardiographic parameters, were well balanced between the early surgery and conservative treatment groups. The mean LVmass index (g/m^2^) in the conservative and early surgery groups was 160 (95%CI: 145–175) and 156 (95%CI: 140–171), respectively (*p* = 0.696). 

Concomitant coronary artery bypass grafting was performed in three patients in the early surgery group and two in the conservative treatment group who required surgery. All other patients underwent aortic valve replacement. Operative mortality in early surgery group was 1.7% (one patient died within 1 month of the surgery). In the conservative treatment group, one patient also died within 30 days of the surgery. 

There was a total of 44 (37.9%) patients with MACEs during follow up. Patients who experienced MACEs were older (*p* = 0.007), more often had DM (*p* = 0.018), had higher STS scores (*p* = 0.003) and did not undergo initial aortic valve replacement (*p* < 0.001). Patients with MACEs had significantly higher mortality rates, both cardiovascular and overall (*p* < 0.001 and *p* < 0.001, respectively). Detailed baseline clinical characteristics of the study population are presented in Table 1.

**Figure 1:**
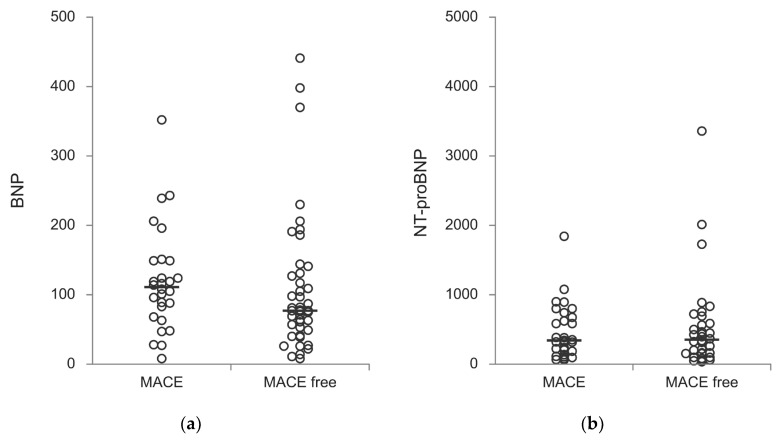
Laboratory parameters of study population between groups: (**a**) BNP (pg/mL) and (**b**) NT-proBNP (pg/mL).

No significant difference in values of BNP and NT-proBNP were found between groups (*p* = 0.107 and *p* = 0.762, respectively) (Figure 1a,b).

Echocardiographic characteristics of the study population according to MACEs are presented in Table 2. Patients with MACEs had lower baseline LVESV (*p* = 0.035), LVEDV (*p* = 0.044) and TAPSE (*p* = 0.029) values. Relative wall thickness was significantly higher in patients with MACEs in contrast to patients who did not experience MACEs (*p* = 0.013).

**Figure 2:**
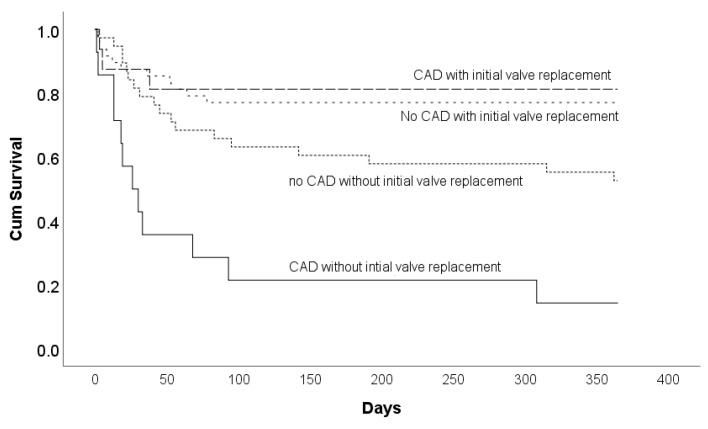
MACE-free survival according to presence of CAD and early surgery for aortic valve replacement.

MACE-free survival according to the presence of CAD and early surgery for aortic valve replacement is presented in Figure 2. One-year MACE-free survival in patients with CAD and early conservative treatment was found to be significantly lower in comparison to that in other patients (Log Rank = 29.062, *p* < 0.001). In patients with no CAD as well as in patients with CAD and early surgery for aortic valve replacement, the median was not reached, while the median MACE-free survival in patients with CAD and early conservative treatment was only 26 days (95% CI for median 5.832 to 46.168 days).

Univariate and multivariate Cox regression analyses for one-year MACE-free survival are shown in Table 3 and Table 4. In univariate Cox regression, significant risk factors for MACE-free survival were identified: the presence of CAD and early conservative treatment (*p* = 0.004), age (*p* = 0.003), the presence of diabetes mellitus (*p* = 0.016) and STS score (*p* = 0.039) (Table 3). According to the multivariate Cox regression analysis, the presence of CAD with early conservative treatment was the most important predictor of MACE-free survival in asymptomatic patients with severe aortic stenosis (*p* ≤ 0.001) (Table 4).

## 4. Discussion

This study incorporates key insights addressing the management of asymptomatic patients with severe aortic stenosis, the prevalence of silent CAD in these patients and their long-term prognosis in terms of MACE-free survival. According to our results, the presence of CAD in patients with early conservative treatment was the most important risk factor for lower MACE-free survival in asymptomatic patients with severe aortic stenosis.

The RECOVERY [7] and AVATAR [4] trials were the first to explore the contentious decision-making process regarding early surgery versus a conservative approach in asymptomatic patients with severe AS and normal left ventricular (LV) function. Traditionally, watchful waiting has been favored due to perceived lower risks of sudden death [7,8,9,10,11], but the trials challenge this by demonstrating that early surgery reduces the incidence of a composite primary outcome, including all-cause death, acute myocardial infarction, stroke and unplanned hospitalization for heart failure (HF) [4].

Expanding on these findings, Banovic et al. [6] introduced a crucial aspect often overlooked—the prevalence of silent CAD in asymptomatic patients with severe AS and normal LVEF. Coronary angiography in this patient population reveals that one in five patients has significant CAD despite the absence of inducible ischemia during exercise testing. This finding challenges the traditional “watchful waiting” strategy, as concomitant severe AS and CAD may lead to more complex interventions, such as simultaneous aortic valve replacement (SAVR) and coronary artery bypass grafting (CABG) or percutaneous coronary intervention (PCI). Our study confirmed the significant prevalence of CAD in asymptomatic patients with severe AS and normal LVEF and, in addition, placed emphasis on the challenge of detecting CAD in asymptomatic AS patients, given the similarity in symptoms and risk factors between the two conditions [12]. Exercise testing is proposed as a potential method to unmask significant CAD according to Banovic et al., but safety concerns and limited sensitivity and specificity complicate its widespread adoption [2,13,14,15]. Furthermore, the importance of non-invasive imaging, such as multi-slice computed tomography coronary angiography, as a proactive screening tool for significant CAD in severe asymptomatic AS patients is usually underscored. This aligns with the notion that silent ischemia, defined as objective evidence of myocardial ischemia without associated chest pain during ET, may be present in a significant proportion of cases [16,17,18].

The role of B-type natriuretic peptide (BNP) and blood glucose as potential biomarkers for CAD are also highlighted in previous studies, with elevated BNP levels associated with inducible ischemia, LV hypertrophy and stable angina pectoris [6,19,20]. These findings suggest a potential role for BNP as an indicator for intervention in asymptomatic AS patients. In our study, BNP was not associated with MACE-free survival, but the presence of diabetes mellitus influenced the long-term survival of these patients. The role of silent ischemia in patients with diabetes was also previously well recognized [21]. 

However, regardless of recommended diagnostic method for the detection of silent CAD in asymptomatic patients with severe AS, the results of our study highlight the significance of coronary artery disease in influencing the decision for surgery, especially when surgical myocardial revascularization is required. The potential risks associated with unrecognized coronary artery disease in patients with hemodynamically significant AS, including ischemia, malignant arrhythmias and adverse cardiovascular events, should be evaluated during decision making about the choice of treatment (invasive or conservative). The nature of our study steps away from Banovic et al. [6], as it extends for a longer period of time, this being the main difference between the two studies. Tracking patients over a one-year period provided further insight into new developments as opposed to tracking them in the short term. In long-term follow up, the results of our study revealed that early surgery for patients with concomitant CAD is beneficial in asymptomatic patients with severe AS. 

### Strengths and Limitations

Crucially, our study points out the lack of clear data on the frequency of latent coronary artery disease in asymptomatic patients with severe AS and normal left ventricular ejection fraction. Additionally, we emphasize a need for comparative data on the frequency of adverse events in asymptomatic patients with severe AS considering the presence or absence of hidden coronary artery disease and choice of treatment—early surgery for aortic valve replacement or conservative therapy. One of the limitations of the study was the lack of cardiac troponin serum concentration measurement, as cardiac troponin serum concentration was shown to be correlated with numerous parameters of subclinical cardiovascular dysfunction [22]. Furthermore, it is important to note the limitations associated with the absence of data regarding the administration of antiplatelet agents and anticoagulants among the participants in our study. 

## 5. Conclusions

In conclusion, our study paints a comprehensive picture of the complexities surrounding the management of asymptomatic severe AS patients, considering both early treatment choices and the prevalence of silent CAD. It emphasizes the need for a nuanced approach, incorporating CAD assessment to make informed decisions in this patient population. Further research and larger studies are warranted to validate these findings and refine treatment strategies in order to adopt a more personalized approach.

## Figures and Tables

**Table 1:** Baseline clinical characteristics of the study population according to MACE occurrence.

Variable	MACE		*p*
	No (n = 72)	Yes (n = 44)	
Sex, n (%)			
Male	28 (38.9)	22 (50.0)	0.241
Female	44 (61.1)	22 (50.0)	
Age (years), mean ± sd	65.4 ± 9.6	70.4 ± 9.1	0.007
STS score (%), median (25th–75th percentile)	1.45 (1.02–2.18)	2.24 (1.29–3.49)	0.003
BMI (kg/m^2^), mean ± sd	27.55 ± 4.40	28.88 ± 3.97	0.162
BSA, mean ± sd	1.95 ± 0.18	1.91 ± 0.14	0.151
Etiology, n (%)			
Degenerative AS	38 (77.6)	33 (91.7)	0.083
Bicuspid AS	11 (22.4)	3 (8.3)	
Heredity, n (%)	3 (4.2)	4 (9.1)	0.289
Comorbidities, n (%)			
Atrial fibrillation	1 (1.4)	0 (0.0)	0.429
Diabetes mellitus	12 (16.9)	16 (36.4)	0.018
Hypertension	64 (91.4)	40 (90.9)	0.924
Smoking, n (%)	2 (2.8)	1 (2.3)	0.981
Initial aortic valve replacement, n (%)	50 (69.4)	14 (31.8)	<0.001
CAD, n (%)	15 (20.8)	15 (34.1)	0.114
Cardiovascular death, n (%)	0 (0.0)	19 (43.2)	<0.001
Overall mortality, n (%)	0 (0.0)	25 (56.8)	<0.001

**Table 2:** Echocardiographic characteristics of the study population according to MACE occurrence.

Variable Mean (95%CI)	MACE		*p*
	No (n = 72)	Yes (n = 44)	
Aorta (cm)	3.54 (3.42–3.67)	3.46 (3.29–3.63)	0.400
LVOT (mmHg)	3.81 (3.16–4.45)	3.94 (3.21–4.66)	0.784
LVESV (mL)	35.76 (30.63–40.9)	27.38 (21.18–33.57)	0.035
LVESD (mL)	3.02 (2.90–3.13)	2.90 (2.73–3.07)	0.246
LVEDV (mL)	117.40 (105.15–129.64)	100.5	0.056
LVEDD (mL)	5.20 (5.08–5.31)	5.0 (4.84–5.16)	0.044
LA (cm)	4.13 (3.97–4.28)	4.09 (3.95–4.23)	0.709
Septum	1.28 (1.24–1.31)	1.28 (1.23–1.34)	0.839
PW	1.14 (1.11–1.18)	1.19 (1.14–1.24)	0.158
LVEF (%)	67.80 (66.01–69.59)	69.44 (66.66–72.22)	0.300
SV	85.25 (77.67–92.83)	84.91 (74.32–95.50)	0.956
SVi (mL/m^2^)	41.63 (38.62–44.64)	41.31 (37.55–45.06)	0.894
LVD mass index (g/m^2^)	157.52 (144.63–170.41)	158.46 (139.50–177.42)	0.931
Relative wall thickness	0.44 (0.43–0.46)	0.48 (0.45–0.50)	0.013
TAPSE (mm)	2.22 (2.13–2.30)	1.94 (1.67–2.22)	0.029
SPDK (mmHg)	31.33 (27.31–35.35)	28.81 (21.75–35.87)	0.492
RV (cm)	2.27 (2.19–2.34)	2.19 (2.09–2.29)	0.208
Vmax (m/s)	4.54 (4.44–4.63)	4.54 (4.44–4.64)	0.966
Pmax (mmHg)	81.94 (78.15–85.72)	82.41 (78.63–86.18)	0.869
Pmean (mmHg)	53.01 (49.61–56.41)	51.17 (48.70–53.64)	0.439
AVA (cm^2^)	0.71 (0.66–0.76)	0.71 (0.66–0.76)	0.960
AVAi (cm^2^/m^2^)	0.36 (0.34–0.39)	0.37 (0.34–0.39)	0.767
Zva (mmHg.mL^−1^.m^2^)	4.83 (4.46–5.20)	4.76 (4.29–5.24)	0.826
ELI	0.45 (0.41–0.48)	0.46 (0.42–0.50)	0.656
SWL	54.68 (50.93–58.44)	54.44 (51.22–57.66)	0.929
AVR	315.10 (281.82–348.37)	295.45 (274.27–316.63)	0.380

**Table 3:** Univariate Cox regression analysis for one-year MACE-free survival.

Variable	Hazard Ratio	95% CI	*p*
No CAD without initial aortic valve replacement	ref		
No CAD with initial aortic valve replacement	0.450	0.212–0.953	0.037
CAD without initial aortic valve replacement	2.958	1.415–6.185	0.004
CAD with initial aortic valve replacement	0.366	0.108–1.242	0.107
Age	1.059	1.019–1.101	0.003
Diabetes mellitus	2.131	1.151–3.945	0.016
STS score	1.147	1.007–1.307	0.039

**Table 4:** Multivariate Cox regression analysis for one-year MACE-free survival.

Variable	Hazard Ratio	95% CI	*p*
No CAD without initial aortic valve replacement	ref		
No CAD with initial aortic valve replacement	0.557	0.238–1.304	0.178
CAD without initial aortic valve replacement	5.472	2.199–13.618	<0.001
CAD with initial aortic valve replacement	0.529	0.151–1.856	0.320

## Data Availability

The data that support the findings of this study are available on request from the corresponding author.
